# Associative Analysis of lncRNA/circRNA-miRNA-mRNA Expression Profiles in Iron-Overloaded HT-1080 Fibrosarcoma Cells

**DOI:** 10.3390/ijms27125617

**Published:** 2026-06-22

**Authors:** Yifan Teng, Qian Zhang, Haoxuan Ding, Jie Feng

**Affiliations:** Key Laboratory of Animal Feed and Nutrition of Zhejiang Province, College of Animal Sciences, Zhejiang University, Hangzhou 310058, China

**Keywords:** iron overload, HT-1080 cells, ceRNA, lncRNA, circRNA, regulatory network

## Abstract

Iron overload disrupts cellular homeostasis and drives ferroptosis through dysregulated iron metabolism. Non-coding RNAs (ncRNAs) are considered as key regulators of various biological functions and targets for a new generation of RNA therapeutics and biomarkers. However, few studies have investigated the regulatory roles of ncRNAs, particularly competitive endogenous RNAs (ceRNAs) in iron overload. This study performed whole-transcriptome sequencing to characterize the ceRNA network in ferric ammonium citrate (FAC)-induced iron-overloaded HT-1080 fibrosarcoma cells. A total of 208 differentially expressed mRNAs, 83 lncRNAs, and 170 circRNAs (*q* < 0.05) were identified, with hierarchical clustering revealing distinct expression patterns between control and iron-treated groups. KEGG enrichment implicated vitamin B6 metabolism (*q* < 0.001) and lysine degradation (*q* < 0.001) as key disrupted pathways. ceRNA network was conducted and further demonstrated lncRNA/circRNA-mediated regulation of ferroptosis genes via shared miRNA response elements. Notably, *LINC-PINT-232* was implicated in the regulation of both ferritin heavy chain (*FTH*) and sequestosome 1 (*SQSTM1*), two ferroptosis-associated mRNAs. *FTH* upregulation mitigates iron toxicity through ferroxidase activity, while *SQSTM1* modulates lipid peroxidation in ferroptosis. These findings provide a preliminary transcriptomic landscape for hypothesis generation regarding ncRNA-mediated regulatory mechanisms in iron overload-induced ferroptosis and offer a computational foundation for future functional and therapeutic investigations.

## 1. Introduction

Iron functions as a critical trace mineral in human biology, participating in fundamental physiological and biochemical processes such as energy metabolism, oxygen transportation, and DNA biosynthesis. The maintenance of iron homeostasis is crucial for human health [1]. However, excessive iron undergoes redox cycling between Fe^3+^ and Fe^2+^, acting as electron carriers to catalyze reactive oxygen species (ROS) production. This process damages macromolecules and impairs mitochondrial and lysosomal organelles [2]. Ferroptosis is a unique iron-dependent form of programmed cell death involved in the occurrence and progression of diverse human diseases, including cancer. Unlike other forms of cell death, ferroptosis is characterized by ROS-induced lipid peroxidation driven by massive iron accumulation. Iron overload is closely associated with diseases such as cardiovascular disorders, neurodegenerative diseases, diabetes, and cancer [3,4].

Non-coding RNAs (ncRNAs) are considered as key regulators of various cellular processes and involve in the molecular regulatory mechanisms of iron metabolism and ferroptosis [5,6,7]. The competing endogenous RNA (ceRNA) hypothesis proposes that mRNAs, lncRNAs, and circRNAs interact in a regulatory network by competing for shared microRNAs (miRNAs) through microRNA response elements (MREs) [8,9,10]. Numerous studies have confirmed the regulatory roles of the ceRNA network in ferroptosis, with ceRNAs progressively emerging as promising targets for ferroptosis-related cancer therapy [11,12]. However, to date, research on the regulatory relationship of ceRNAs in the context of iron overload remains relatively limited, particularly regarding lncRNAs and circRNAs. As key regulators of ferroptosis, the latent regulatory roles of lncRNAs and circRNAs in iron-overloaded cells may directly or indirectly contribute to ferroptosis. Elucidating these mechanisms holds significant implications for developing targeted therapies against ferroptosis.

In this study, we employed ferric ammonium citrate (FAC) to induce iron overload in HT-1080 fibrosarcoma cells (HT-1080 cells), generating whole-transcriptome profiles of mRNAs, lncRNAs, and circRNAs to construct a regulatory network related to iron overload involving lncRNAs/circRNAs, miRNAs, and mRNAs. This integrative study provides a bioinformatics foundation for transcription-level insights into iron overload mechanisms.

## 2. Results

### 2.1. Sequencing Data Analysis

After 24 h treatment with 4 mM FAC, HT-1080 cells were lysed for total RNA extraction. RNA concentration in the control group (C1–C6) and treatment group (F1–F6) ranged from 452 to 560 ng/μL. Agilent integrity values (9.5–9.9) of all samples met requirements for subsequent library construction and sequencing (Table S1). Additionally, 1% agarose gel electrophoresis showed clear 28S and 18S bands for all 12 samples, confirming RNA purity (Figure S1). Among the 12 samples, six with the highest integrity values (C2, C5, C6, F1, F2, and F5) were selected for library preparation and sequencing, and renamed as CO1, CO2, CO3, IO1, IO2, and IO3. Sequencing error rates at each nucleotide position were 0.02–0.03% in both CO and IO groups, meeting quality requirements (Figure S2a,b).

### 2.2. Quantitative Analysis of lncRNAs and mRNAs

**Figure 1:**
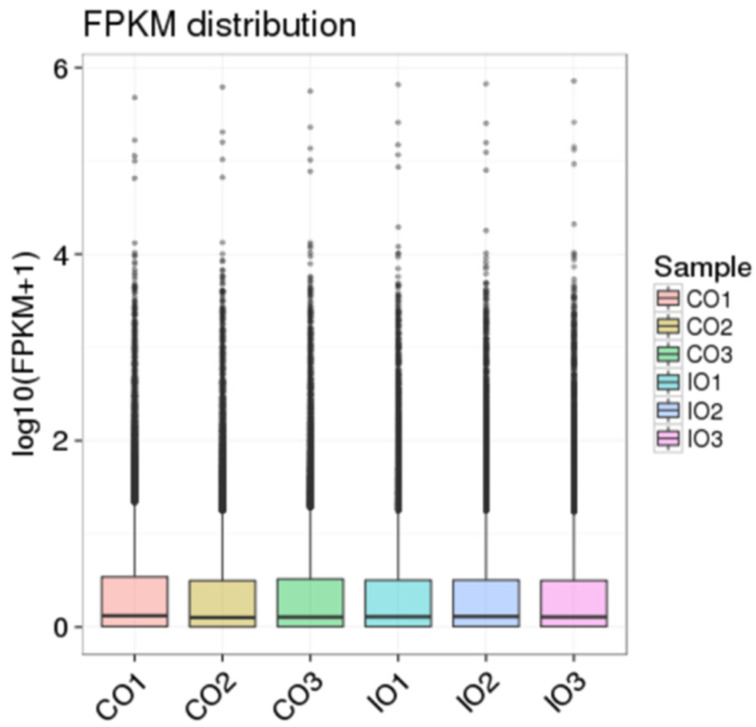
Expression level distributions across samples.

The StringTie network flow algorithm was applied for transcript and gene assembly and quantification. After FPKM calculation, box plots were generated to display expression level distributions of genes or transcripts across samples (Figure 1). Overall, expression levels exhibited consistency between groups, with a slight upward trend observed in the IO group compared to controls.

### 2.3. Differential Expression Analysis of mRNAs/lncRNAs/circRNAs

#### 2.3.1. Differential Analysis of mRNAs

**Figure 2:**
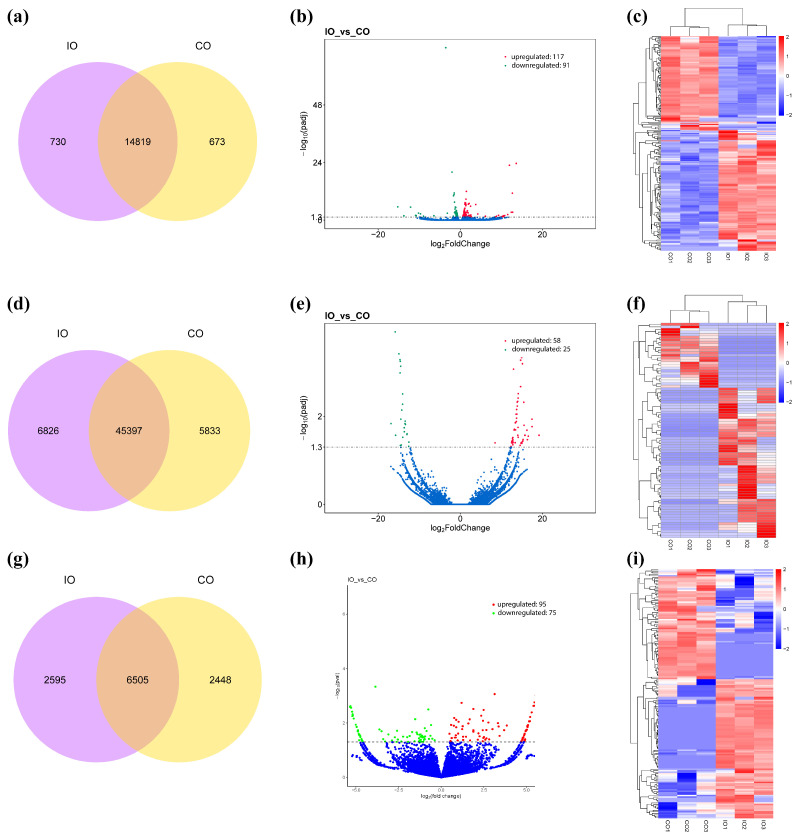
Differentially expressed mRNAs/lncRNAs/circRNAs (DE-mRNAs/lncRNAs/circRNAs). (**a**) Venn plot of DE-mRNAs. (**b**) Volcano plot of DE-mRNAs. (**c**) Hierarchical clustering heatmap of DE-mRNAs. Red indicates upregulation, and blue indicates downregulation. (**d**) Venn plot of DE-lncRNAs. (**e**) Volcano plot of DE-lncRNAs. (**f**) Hierarchical clustering heatmap of DE-lncRNAs. (**g**) Venn plot of DE-circRNAs. (**h**) Volcano plot of DE-circRNAs. (**i**) Hierarchical clustering heatmap of DE-circRNAs. Red dots indicate upregulated DEGs, green dots represent downregulated genes, and blue dots denote genes with no significant change.

Based on the expression matrix obtained from quantitative analysis, differential expression (DE) gene analysis was performed. Iron treatment induced 14,819 shared mRNAs between the CO and IO groups, with 673 mRNAs unique to the CO group and 730 exclusive to the IO group (Figure 2a). As shown in the volcano plot (Figure 2b), 117 genes were significantly upregulated and 91 genes downregulated in the IO group compared to the CO group. Subsequently, with results visualized in a hierarchical clustering heatmap (Figure 2c), the top five significantly upregulated-DE and downregulated-DE mRNAs are shown in Table 1 and Table 2, where *GRIK2* exhibited the highest fold-upregulation (*q* < 0.0001) and *AL121900.2* showed the highest fold-downregulation (*q* < 0.0001).

#### 2.3.2. Differential Analysis of lncRNAs

lncRNAs regulate target gene expression at both transcriptional and post-transcriptional levels, primarily through co-location and co-expression mechanisms. Iron treatment induced 45,397 shared lncRNAs between the control and IO groups, with 5833 lncRNAs unique to the CO group and 6826 exclusive to the IO group (Figure 2d). Volcano plots visualized the DE-lncRNAs (Figure 2e), with 58 significantly upregulated and 25 significantly downregulated in the IO group compared to the CO group. Further, hierarchical clustering results revealed differences between IO and CO groups (Figure 2f). The top five significantly upregulated and downregulated lncRNAs are shown in Table 1 and Table 2, where *GUSBP1-202* exhibited the highest fold-upregulation (*q* < 0.05) and *ZEB1-223* showed the highest fold-downregulation (*q* < 0.05).

#### 2.3.3. Differential Analysis of circRNAs

circRNA has a length greater than 200 nt and is primarily generated from pre-mRNA through alternative splicing. Iron treatment induced 6505 shared circRNAs between the control and IO groups, with 2448 circRNAs unique to the CO group and 2595 exclusive to the IO group (Figure 2g). Then, we performed differential expression analysis of circRNAs using DESeq2 (version 1.24.0) with *p* < 0.05 as the filtering criterion. Volcano plots revealed 95 significantly upregulated and 75 downregulated circRNAs in the IO group compared to controls (Figure 2h). Hierarchical clustering analysis was conducted to evaluate expression patterns of DE-circRNAs, with clustering results visualized in Figure 2i. The top five significantly upregulated and downregulated lncRNAs are shown in Table 1 and Table 2, where *novel_circ_0001438* exhibited the highest fold-upregulation (*q* < 0.0001) and *hsa_circ_0004276* showed the highest fold-downregulation (*q* < 0.05).

#### 2.3.4. Validation of DE-mRNAs/lncRNAs/circRNAs

We randomly selected DE-mRNAs, DE-lncRNAs and DE-circRNAs to verify the validity of RNA sequencing using qRT-PCR. According to the results of qRT-PCR, the selected DE-mRNAs/lncRNAs/circRNAs exhibited significant differences, consistent with the trend of the sequencing results (Figure S3a–f; *p* < 0.05). Overall, the qRT-PCR results confirmed the reliability of the RNA sequencing data.

### 2.4. Functional Analysis of mRNAs/circRNAs

#### 2.4.1. GO and KEGG Enrichment Analysis of DE-mRNAs

**Figure 3:**
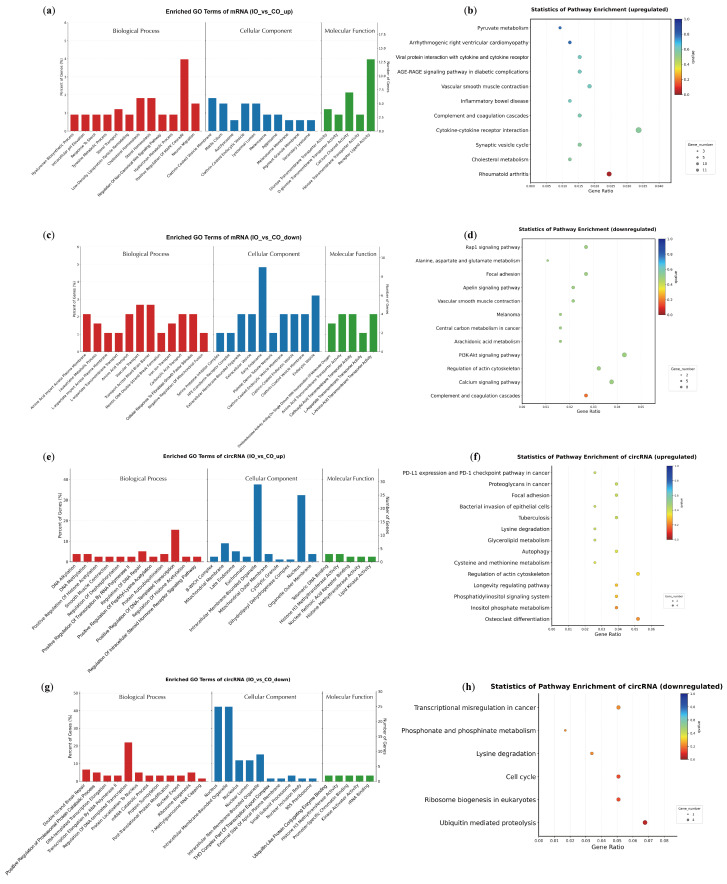
mRNA/circRNA gene ontology (GO) and Kyoto Encyclopedia of Genes and Genomes (KEGG) enrichment analysis diagram. (**a**) Upregulated mRNA GO enrichment bar chart. (**b**) KEGG enrichment analysis of upregulated mRNAs. (**c**) Downregulated mRNA GO enrichment bar chart. (**d**) KEGG enrichment analysis of downregulated mRNAs. (**e**) Upregulated circRNA GO enrichment bar chart. (**f**) KEGG enrichment analysis of upregulated circRNAs. (**g**) Downregulated circRNA GO enrichment bar chart. (**h**) KEGG enrichment analysis of downregulated circRNAs. Red represents biological process (BP), blue represents cellular component (CC), and green represents molecular function (MF). The diameter of a point represents the number of target genes enriched in a specific item, and the adjusted *q*-value represents the degree of enrichment.

GO enrichment analysis of upregulated mRNAs (Figure 3a) revealed that biological process (BP) terms were predominantly associated with positive regulation of MAPK cascade, cholesterol homeostasis, and sterol homeostasis. Cellular component (CC) enrichment was mainly related to clathrin-coated vesicle membranes, motile cilia, and lysosomal lumen. Molecular function (MF) analysis showed that receptor ligand activity, glucose transmembrane transporter activity, and calcium channel activity were the most significantly enriched terms. KEGG enrichment analysis of upregulated mRNAs (Figure 3b) identified 11 significantly enriched pathways, with rheumatoid arthritis being the most significantly enriched pathway (*q* value < 0.05), followed by cholesterol metabolism and synaptic vesicle cycle. Other enriched pathways included cytokine–cytokine receptor interaction, which aligned with the inflammatory response induced by iron overload.

GO enrichment analysis of downregulated mRNAs (Figure 3c) showed that BP terms were predominantly associated with amino acid import across the plasma membrane, vascular transport, and transport across blood–brain barrier. CC enrichment highlighted the early endosome and endocytic vesicle. MF terms were mainly enriched in oxidoreductase activity and amino acid transmembrane transporter activity. KEGG enrichment analysis of downregulated mRNAs (Figure 3d) identified 12 significantly enriched pathways. Complement and coagulation cascades represented the most significantly enriched pathway, followed by calcium signaling and regulation of actin cytoskeleton.

#### 2.4.2. GO and KEGG Enrichment Analysis of DE-circRNAs

For upregulated circRNAs (Figure 3e,f), GO analysis revealed that BP terms were predominantly associated with DNA methylation, DNA alkylation, and positive regulation of histone acetylation. CC enrichment identified the intracellular membrane-bounded organelle and nucleus as the primary components. MF terms were enriched in telomeric DNA binding and histone H3 methyltransferase activity, suggesting that upregulated circRNAs may be involved in epigenetic regulation. KEGG enrichment analysis identified 14 significant pathways, among which osteoclast differentiation and inositol phosphate metabolism were the most enriched.

For downregulated circRNAs (Figure 3g,h), GO analysis revealed that BP terms were predominantly enriched in double-strand break repair, DNA-templated transcription elongation, and positive regulation of proteasomal protein catabolic process. CC terms were mainly associated with the nucleus, intracellular membrane-bounded organelles, and the nucleolus. MF enrichment included ubiquitin-like protein conjugating enzyme binding and histone H3 methyltransferase activity. KEGG enrichment analysis identified six significant pathways, with ubiquitin-mediated proteolysis being the most significantly enriched, followed by ribosome biogenesis in eukaryotes and cell cycle.

### 2.5. lncRNA/circRNA-miRNA-mRNA Interaction Network Analysis

**Figure 4:**
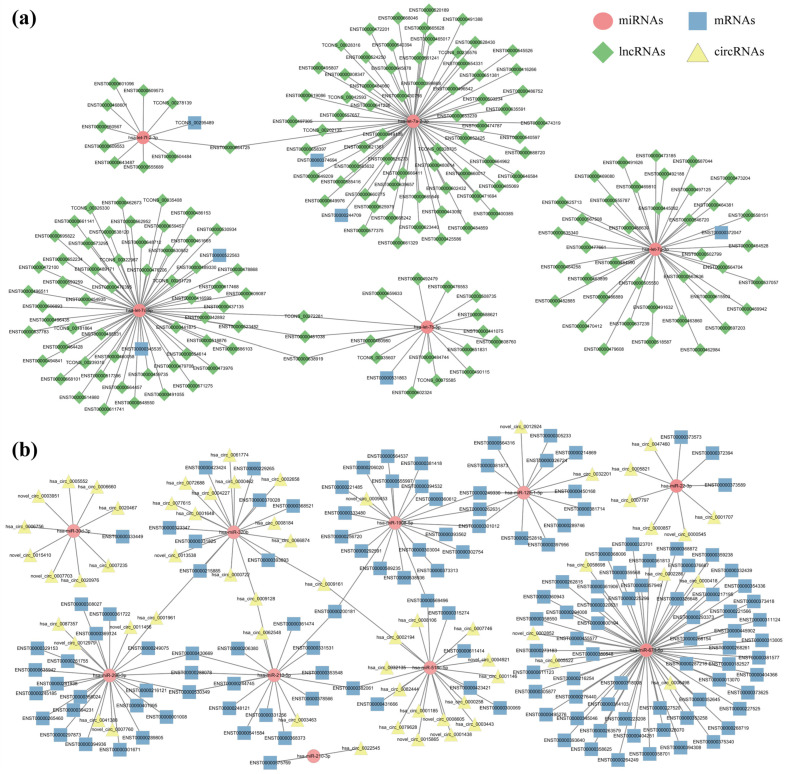
lncRNA/circRNA-miRNA-mRNA network interaction diagram. (**a**) lncRNA-miRNA-mRNA network interaction diagram. (**b**) circRNA-miRNA-mRNA network interaction diagram. Red (miRNAs), blue (mRNAs), green (lncRNAs), and yellow (circRNAs).

We calculated Pearson correlation coefficients between miRNAs and their targeting mRNAs (threshold: negative correlations). Analysis identified 6053 miRNA-mRNA pairs with significant inverse associations (*r* < 0, *p*adj < 0.05). Then, we calculated Pearson correlation coefficients between miRNAs and their targeting lncRNAs. A total of 10,110 lncRNAs exhibited significant negative correlations with associated miRNAs (*r* < 0, *p*adj < 0.05). Finally, we computed Pearson correlation coefficients between miRNAs and their targeting circRNAs. A total of 10,310 circRNAs showed significant negative correlations (*r* < 0, *p*adj < 0.05). Then, we constructed the lncRNA/circRNA-miRNA-mRNA interaction network (Figure 4a). The top 200 miRNAs were screened for target mRNAs (negatively correlated expression) and target circRNAs (negatively correlated expression). The interaction network of circRNAs-miRNAs-mRNAs is shown in Figure 4b.

### 2.6. Functional Analysis of lncRNA/circRNA-miRNA-mRNA Regulatory Networks

#### 2.6.1. GO Enrichment Analysis of lncRNA-miRNA-mRNA Regulatory Networks

**Figure 5:**
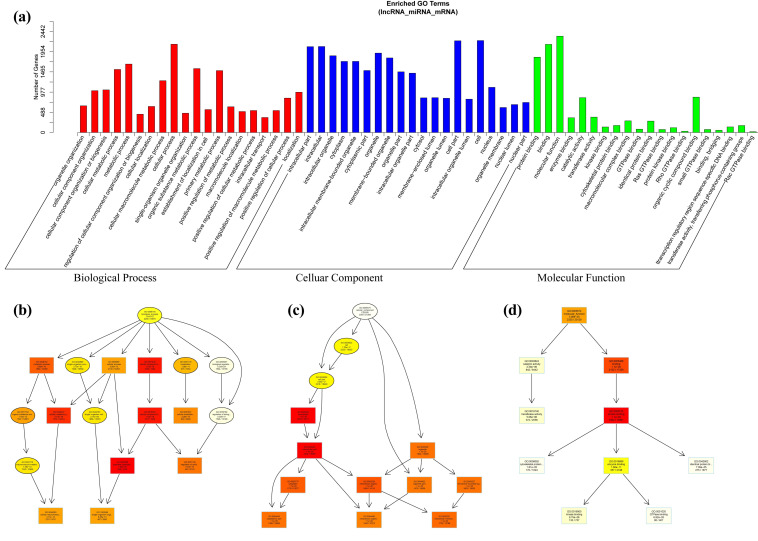
GO enrichment analysis of lncRNA-miRNA-mRNA regulatory networks. (**a**) Scatter plot of source genes. (**b**–**d**) Directed acyclic graphs (DAGs) illustrating hierarchical relationships of enriched GO terms. Branches denote inclusion relationships, with functional specificity increasing from top to bottom. Rectangular nodes represent the top 10 significantly enriched terms, while node color intensity reflects the degree of enrichment (darker indicates lower *p*-values). Each node displays the GO ID, description, *p*-value, and the ratio of target genes to background genes.

We performed GO analysis on the mRNAs within the ceRNA network to explore their potential biological functions. Enriched BP categories primarily included cellular processes, metabolic processes, and cellular metabolic processes (Figure 5a). CC predominantly involved intracellular elements, intracellular parts, cells, and cell parts. MF categories enriched in target genes were protein binding, binding, and molecular function. Based on GO enrichment results, the top 10 target gene enrichment outcomes were selected as primary nodes in the directed acyclic graph (DAG) to visualize hierarchical functional relationships. Among biological processes, cellular component organization or biogenesis demonstrated the highest downstream enrichment significance (Figure 5b). Within cellular components, ‘intracellular’ and ‘intracellular part’ showed high enrichment significance and are hierarchically contained within the broader category ‘cell part’ (Figure 5c). Among molecular functions (Figure 5d), ‘binding’ emerged as the dominant category, encompassing the majority of functional annotations in target genes.

#### 2.6.2. GO Enrichment Analysis of circRNA-miRNA-mRNA Regulatory Network

**Figure 6:**
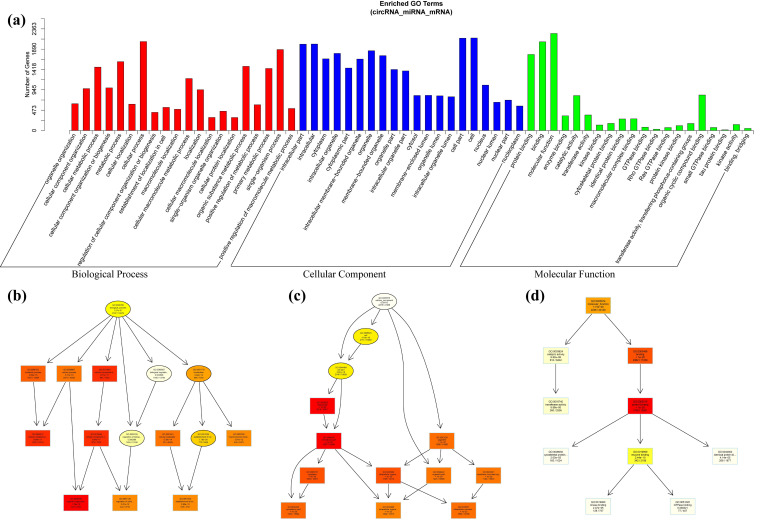
GO enrichment analysis of circRNA-miRNA-mRNA regulatory networks. (**a**) Scatter plot of source genes. (**b**–**d**) DAG of circRNA-miRNA-mRNA regulatory network GO enrichment analysis.

GO functional enrichment analysis of target mRNAs genes was performed, and results are presented as a GO functional enrichment bar chart. Enriched biological process categories were predominantly associated with cellular processes, single-organism processes, and metabolic processes (Figure 6a). In line with previous lncRNA-miRNA-mRNA association analysis, cellular components were predominantly associated with intracellular, intracellular part, cell, and cell part. Additionally, molecular functions of enriched target genes were dominated by protein binding, binding, and molecular function. Based on GO enrichment analysis, the top 10 enriched target gene categories were selected as primary nodes in the DAG to visualize hierarchical functional relationships. In biological processes, ‘cellular component organization or biogenesis’ and its downstream processes, along with ‘cellular metabolic processes’, exhibited higher enrichment significance (Figure 6b). Enrichment patterns for cellular components and molecular functions were consistent with results from the lncRNA-miRNA-mRNA association analysis (Figure 6c,d).

### 2.7. Construction of lncRNA/circRNA-miRNA–Ferroptosis-Related mRNA Network

**Figure 7:**
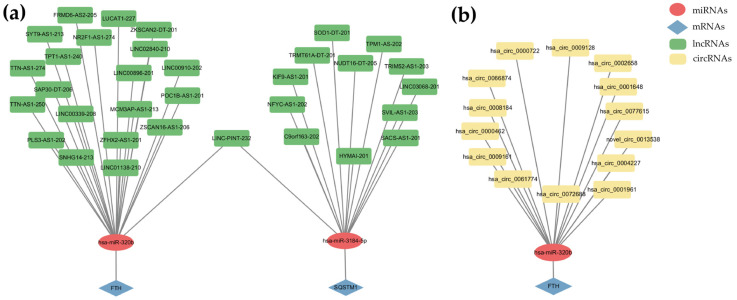
lncRNA/circRNA-miRNA–ferroptosis-related mRNA network interaction diagram. Red (miRNAs), blue (mRNAs), green (lncRNAs), and yellow (circRNAs). (**a**) lncRNA/circRNA-miRNA–ferroptosis-related mRNA network. (**b**) circRNA-miRNA–ferroptosis-related mRNA network.

The FerrDb database contains 487 ferroptosis-related mRNAs. After aligning these 487 ferroptosis-related mRNAs with the previously mentioned 208 DE-mRNAs, 2 significantly upregulated mRNAs were identified: ferritin heavy chain (*FTH*) and sequestosome 1 (*SQSTM1*). Next, we screened lncRNAs, circRNAs, and miRNAs that exhibit targeting relationships with ferroptosis-related mRNAs and are negatively correlated in expression, then constructed lncRNA/circRNA-miRNA–ferroptosis-related mRNA regulatory interaction networks. Among these, the lncRNA-miRNA–ferroptosis-related mRNA network involved 34 lncRNAs and 2 miRNAs (Figure 7a), while another lncRNA-miRNA–ferroptosis-related mRNA network involved 14 lncRNAs and 1 miRNA (Figure 7b). Notably, *LINC-PINT-232* was implicated in the regulation of both *FTH* and *SQSTM1*, two ferroptosis-associated mRNAs.

## 3. Discussion

Iron is an essential trace element in biological systems, participating in numerous biological reactions and pathways [13,14]. However, primary and secondary iron overload can occur due to gene mutations in key elements regulating iron homeostasis or accumulation of bodily iron attribute to physiological or pathological factors. Excess iron participates in electron transfer via the mitochondrial oxidative respiratory chain, generating large amounts of ROS, which lead to mitochondrial dysfunction, oxidative stress, lipid peroxidation, and DNA damage. Iron overload is also a major driver of ferroptosis [3]. Iron overload can cause toxic accumulation in the liver, heart, joints or endocrine glands [13]. However, there is currently a lack of ceRNA-related studies focusing on iron overload. Therefore, this study aims to identify DE-mRNAs, DE-lncRNAs, and DE-circRNAs in iron-overloaded HT-1080 cells using transcriptome sequencing and bioinformatics analysis, construct a ceRNA regulatory network, and perform associative analysis with ferroptosis-related genes.

In this study, we established an iron overload model in HT-1080 cells induced by FAC. Then, ribosomal RNA depletion, random fragmentation, and size selection were performed to obtain all RNA species excluding rRNAs and small RNAs, primarily including lncRNAs, mRNAs, and circRNAs during library construction. Quality control of sequencing data is critical for the reliability of downstream analyses. After alignment analysis, 92.24–93.18% of effective data mapped to the reference genome, with reads predominantly concentrated in exonic regions (75.5–77.86%), indicating mRNA as the dominant RNA type in sequencing data. Following transcript assembly, filtering, and prediction workflows, we obtained novel lncRNAs and novel mRNAs. Comparative analysis of transcript length, exon count, and open reading frame (ORF) length revealed distinct features between novel and known RNA species. Novel mRNAs and mRNAs exhibited longer transcript lengths than novel lncRNAs and lncRNAs, consistently with previous reports [15]. Transcript length showed positive correlations with exon number and ORF length, where higher exon counts and longer ORFs indicate robust protein-coding capacity. While lncRNAs lack protein-coding potential, they regulate target gene expression at transcriptional, translational, and epigenetic levels [16]. The reduced exon counts and ORF lengths in novel lncRNAs/lncRNAs compared to mRNAs likely reflect their enrichment in intergenic regions, which inherently harbor non-coding RNAs. Furthermore, intron retention in novel lncRNAs/lncRNAs shortens ORF post-splicing, further restricting protein-coding potential [17]. These comparative analyses validate that the predicted novel lncRNAs and mRNAs conform to canonical features, enabling their inclusion in downstream quantitative studies.

We quantified transcript expression levels for known transcripts, predicted novel lncRNAs, novel mRNAs, and unclassified transcripts using the StringTie network flow algorithm. Despite overlapping expression distributions, 208 mRNAs exhibited significant changes. Iron metabolism-related mRNAs were markedly altered under iron overload, including the downregulation of transferrin receptor (*TFRC*) and Solute Carrier Family 11 Member 2 (*SLC11A2*), and the upregulation of ferritin light chain (*FTL*) and *FTH*. Previous studies demonstrate that iron overload disrupts IRP-IRE interactions, leading to *TFRC* mRNA destabilization and degradation [18,19], which is consistent with our observed *TFRC* downregulation. *SLC11A2* not only mediates intestinal iron absorption but also facilitates non-transferrin-bound iron (NTBI) uptake in organs [20]. Mammalian ferritin, composed of 24 subunits, is critical for iron homeostasis and overload prevention. Glutamate receptor ionotropic kainate 2 (GRIK2), the most significantly upregulated mRNA, has been reported as a novel epigenetic target in gastric cancer [21], While its specific role in our model remains undetermined, we speculate that its severe dysregulation under iron overload could contribute to pro-carcinogenic pathways. D-aminoacyl-tRNA deacylase (*DTD*), a bacterial/eukaryotic tRNA editing factor that removes mischarged D-amino acids and non-canonical glycine from tRNAs [22], was the most downregulated transcript, potentially impairing translation. Collectively, these results reveal widespread mRNA-level diversity following iron treatment.

GO and KEGG enrichment analyses of upregulated and downregulated mRNAs revealed distinct functional landscapes under iron overload. Upregulated mRNAs were primarily enriched in the positive regulation of MAPK cascade, cholesterol homeostasis, and sterol homeostasis. MAPK pathways, particularly ERK and p38, have been reported to modulate ferroptosis sensitivity by influencing RAS–RAF–MEK signaling and oxidative stress responses in cancer cells [23]. In the KEGG analysis, rheumatoid arthritis was the most significantly enriched KEGG term among upregulated mRNAs, reflecting shared inflammatory and iron-dysregulation features between iron overload and arthritis pathogenesis. A previous study has shown that the inhibition of oxidative stress-induced ferroptosis can alleviate rheumatoid arthritis in humans [24]. At present, the inner link between ferroptosis and rheumatoid arthritis has not been studied deeply, but ferroptosis may be a novel strategy for rheumatoid arthritis treatment. In contrast, downregulated mRNAs were predominantly associated with amino acid import across the plasma membrane, vascular transport, and transport across the blood–brain barrier, suggesting broad suppression of membrane transport functions under iron-induced oxidative stress.

A total of 83 lncRNAs displayed significant differential expression. Following target gene prediction, structural variation analysis was performed. rMATS analysis revealed exon skipping as the predominant alternative splicing event with five types post-iron treatment. In hereditary hemochromatosis patients with *HFE* mutations, the c.340+4C splice variant may induce exon 2 skipping via alternative splicing, exacerbating iron overload risk [25].

The majority of identified circRNAs originated from exon splicing and were predominantly located on chromosomes 1–3. Exon skipping represents a key mechanism for exon-derived circRNAs formation [26,27], where iron overload may promote linear RNAs degradation while preserving circRNAs with stable closed-loop structures. Following iron treatment, a total of 2595 DE-circRNAs were identified, and 170 of them showed significant expression changes as visualized by the volcano plot. Previous study demonstrated that circ_0001438 exacerbates human trophoblast dysfunction by mediating the *miR-942/NLRP3* axis [28]. In this study, circ_0001438 was the most significantly upregulated circRNA post-iron overload, suggesting its role in disrupting cellular homeostasis. circ_0004276 was the most significantly downregulated circRNA following iron overload; however, previous studies reported notable upregulation of this circRNA in vertebral tissues of glucocorticoid-induced osteoporosis patients [29], while its functional significance remains uncharacterized. Using circRNA-source gene associations, we performed GO and KEGG enrichment analysis. Unlike small RNAs, lncRNAs, or mRNAs, the enriched pathways for circRNAs predominantly mapped to biological processes and cellular components. GO analysis of upregulated and downregulated circRNAs revealed distinct epigenetic and genomic response programs. Upregulated circRNA hosts were primarily enriched in biological processes including DNA methylation, DNA alkylation, and positive regulation of histone acetylation, with molecular function terms highlighting telomeric DNA binding and histone H3 methyltransferase activity. These findings may demonstrate that iron overload drives transcriptional reprogramming through epigenetic mechanisms, which is consistent with previous evidence that dietary iron overload can cause changes in the methylation of the ferroptosis regulatory factor Nrf2, thereby regulating the occurrence of ferroptosis [30]. Similarly, brain iron overload disrupts the cellular redox environment and impairs global DNA methylation [31].

In contrast, downregulated circRNAs were enriched in double-strand break repair, DNA-templated transcription elongation, and positive regulation of proteasomal protein catabolic process, with cellular components primarily mapped to the nucleus and nucleolus. The suppression of double-strand break repair pathways under iron overload conditions is consistent with the well-established ability of ROS to induce DNA damage while simultaneously impairing DNA repair machinery, thereby promoting genomic instability [32]. Collectively, the enrichment patterns of circRNA host genes suggest that iron overload engages epigenetics reprogramming and compromises genome maintenance mechanisms, potentially contributing to pro-tumorigenic transcriptomic shifts.

Based on negative Pearson correlation coefficients, we predicted miRNA-lncRNA and mRNA-circRNA targeting relationships. Following target prediction, we integrated the expression profiles of the top 200 lncRNAs, miRNAs, and mRNAs; however, limited intermolecular associations were observed among these candidates, with no discernible ceRNA network identified. Additionally, GO functional enrichment analyses were conducted for lncRNA, miRNA, and mRNA datasets. ceRNA networks emerge through RNA interactions mediated by shared miRNA response elements (MREs), where miRNAs act as central regulatory hubs in the lncRNA-miRNA-mRNA tripartite regulatory framework [33]. By integrating the expression profiles of the top 200 circRNAs, miRNAs, and mRNAs, we observed that numerous circRNAs may act as ceRNAs to regulate mRNAs such as *TREM1* (Triggering Receptor Expressed Myeloid Cells 1), *FZD8* (Frizzled Class Receptor 8), and *DPP3* (Dipeptidyl Peptidase 3). A previous study emphasized the interplay between iron, bacteria, and cancer, particularly iron overload’s role in promoting ovarian cancer precursor lesions [34], and demonstrated that transfusion-induced iron overload exacerbates acute myeloid leukemia progression. These findings suggest that iron overload disrupts circRNA-miRNA-mRNA regulatory networks, accelerating carcinogenesis. Additionally, chronic iron overload leads to the accumulation of dysfunctional autolysosomes and the loss of free lysosomes in skeletal muscle, thereby causing insulin resistance due to autophagic defects [35]. However, the specific relationship between ceRNAs and lysosomal dysfunction during iron overload remains unexplored.

Finally, by aligning the DE-mRNAs with ferroptosis-related genes in the FerrDb database and constructing the ceRNA-miRNA–ferroptosis-related mRNA regulatory interaction network, we identified two ferroptosis-related mRNAs: *FTH* and *SQSTM1*. FTH possesses ferroxidase activity, which converts Fe^2+^ to Fe^3+^, thereby storing excess cellular iron within the ferritin nanocage and preventing iron overload-mediated ROS production and tissue damage [36]. *SQSTM1* is a transport protein receptor in autophagy that also participates in multiple molecular pathways, such as activating cysteinyl aspartate specific protease 8 (caspase-8) to regulate apoptosis [37]. Numerous studies have confirmed its critical role in inducing ferroptosis; *SQSTM1* mediates Clockophagy to promote lipid peroxidation during ferroptosis [38], and it can also drive ferroptosis through its downstream effector acyl-CoA synthetase long-chain family member 4 (*ACSL4*) [39]. We noted that the lncRNA *LINC-PINT-232* was involved in both the *FTH* and *SQSTM1* regulatory networks and was negatively correlated with their mRNA expression. *LINC-PINT* is a p53-induced lncRNA that plays significant roles in various diseases, particularly cancers. For instance, overexpression of *LINC-PINT* inhibits gastric tumor growth by downregulating hypoxia-inducible factor-1α (*HIF-1α*), and it suppresses lung cancer progression by sponging miR-543 and inducing phosphatase and tensin homolog [40]. Therefore, given the known tumor-suppressive roles of *LINC-PINT*, its significant downregulation observed in our iron-overload model raises the possibility of a theoretical link between excessive iron stress and pro-tumorigenic transcriptomic shifts. Several RNA-based therapeutics, including antisense oligonucleotides (ASOs) and small interfering RNAs (siRNAs), have gained FDA approval over the past decade. Building on their potent regulatory effects, lncRNA-based therapeutics are of emerging clinical significance. Consequently, targeting the ncRNA–ferroptosis axis is increasingly demonstrating substantial promise as a novel strategy for cancer treatment. Translating this identified axis into clinical therapy primarily centers on targeted expression modulation. Potential strategies include modulating lncRNA (e.g., *LINC00551*) transcription by altering its promoter activity to induce tumor cell ferroptosis [41], or employing ASOs to silence oncogenic sponges that inhibit ferroptosis [42].

However, our study has several limitations. The current transcriptomic profiling was conducted exclusively in the HT-1080 fibrosarcoma cell line. While this provided a robust and specific model for mapping the iron overload-induced ceRNA network, the biological responses to iron toxicity can exhibit cell-type specificity. Therefore, future studies should aim to validate these crucial ncRNA-mRNA interactions, such as the *LINC-PINT-232/FTH/SQSTM1* axis, across multiple cancer cell lines to fully determine their broader physiological relevance and translational potential.

## 4. Materials and Methods

### 4.1. Cell Lines and Cell Culture

The HT-1080 cell line used in this study was obtained from the American Type Culture Collection (ATCC Accession Number: CCL-121, Manassas, VA, USA). Cells were cultured in Dulbecco’s Modified Eagle Medium/Nutrient Mixture F-12 (DMEM/F-12, Gibco, Carlsbad, CA, USA), supplemented with 10% fetal bovine serum (FBS, Gibco, Carlsbad, CA, USA) and 1% penicillin/streptomycin (Pen-Strep, Sigma, St. Louis, MO, USA) at 37 °C in a 5% CO_2_ atmosphere.

### 4.2. Cell Sequencing Sample Collection

HT-1080 cells were seeded into 6-well plates at a density of 2 × 10^5^ cells per well and cultured for 24 h. The control group was treated with complete medium, while the treatment group received 4 mM FAC-containing medium for 24 h. After washing with PBS, 1 mL TRIzol (Invitrogen, Carlsbad, CA, USA) was added to each well. The cells were thoroughly lysed by repeated pipetting to ensure TRIzol contact with all cell-coated surfaces. The digested cell lysate was transferred to 1.5 mL RNase-free centrifuge tubes and vortexed until no visible cell clumps remained, achieving a clear and non-viscous solution. Samples were stored at −80 °C.

### 4.3. Total RNA Extraction and Quality Control

Total RNA was extracted using standard protocols. RNA concentration was quantified using Nanodrop spectrophotometry (Thermo Fisher Scientific, Wilmington, DE, USA). RNA integrity was evaluated with the Agilent 2100 Bioanalyzer (Agilent Technologies, Santa Clara, CA, USA), while RNA purity was assessed through agarose gel electrophoresis and Agilent 2100 analysis. Only samples meeting quality criteria for sequencing libraries were used to construct lncRNA, mRNA, and circRNA libraries.

### 4.4. mRNA, lncRNA and circRNA Sequencing Analysis

Specific libraries were constructed using ribosomal RNA depletion and linear RNA removal methods. Total RNA was first subjected to rRNA and linear RNA depletion, followed by fragmentation into 250–300 bp segments. The fragmented RNA was then used as a template for synthesizing the first strand of cDNA, with subsequent synthesis of the second cDNA strand using dNTPs. Purified double-stranded cDNA underwent end repair, sequencing adapter ligation, and size selection to obtain 350–400 bp cDNA fragments. The USER enzyme was employed to degrade the uracil-containing second cDNA strand, followed by PCR amplification to generate the final library. For circRNA library preparation, a similar workflow was applied, with size selection ensuring the exclusion of ribosomal RNAs and small RNAs (miRNAs, siRNAs, etc.), while retaining lncRNAs, mRNAs, and circRNAs. After library quality control, normalized and pooled samples were sequenced on an Illumina PE150 platform (NEB, Ipswich, MA, USA) based on effective concentration and data output requirements. Initial assessments were conducted for sequencing error rates, data volume, alignment rates, and other quality metrics after obtaining sequencing data. Data mining proceeded only if the results met predefined quality standards.

In this study, we employed a combined approach using find_circ and CIRI for circRNAs identification. Anchor sequences of 20 nucleotides (nt) were extracted from both ends of reads that failed to align to the reference genome. These sequences were then realigned to the reference genome. If the 5′ end of the anchor sequence aligned successfully, the 3′ end was repositioned upstream of the original alignment site. A circRNA was identified if splicing junctions were detected at both the start and end positions, with a read count ≥ 2. For CIRI, junction reads were identified by scanning PCC signals in SAM files, followed by filtering using PEM and GT-AG signals, and finally detecting unbalanced junction reads based on the DM algorithm.

### 4.5. GO and KEGG Enrichment Analysis

To capture the overarching systemic biological responses, gene ontology (GO) and Kyoto Encyclopedia of Genes and Genomes (KEGG) enrichment analyses were performed collectively on all differentially expressed genes (distinguishing between upregulated and downregulated subsets). The functional enrichment was conducted using the clusterProfiler package (version 4.6.2) and enrichplot package (version 1.18.4) in R (version 4.5.1), with a *p*-value < 0.05 and a *q*-value < 0.05 considered statistically significant.

### 4.6. ceRNA Regulation Analysis

The ceRNA hypothesis proposes that non-coding RNAs containing miRNA-binding sites can regulate mRNA expression levels by competitively binding to and forming RISC (RNA-induced silencing complex). Guided by this hypothesis, we constructed a miRNA-centric intracellular regulatory network by aggregating the relative expression relationships of various ncRNAs and mRNAs. To control for multiple testing in the correlation analysis, Benjamini–Hochberg false discovery rate (FDR) correction was applied to all Pearson correlation *p*-values using the p.adjust() function in R (version 2026.01.2). Only interaction pairs meeting the criteria of |*r*| > 0.8 and FDR-adjusted *p* < 0.05 were retained for network construction. We screened the top 200 miRNAs and identified their target mRNAs and lncRNAs with significantly negatively correlated expression profiles. The lncRNA/circRNA-miRNA-mRNA interaction networks were visualized using Cytoscape (v3.10.3). To identify key regulatory hubs, we performed degree centrality analysis, where the degree of each node was calculated based on the number of its direct connections. In the resulting network diagrams, node size was mapped to the degree value, highlighting ‘hub’ molecules that possess the highest regulatory influence.

## 5. Conclusions

This study delineated the ceRNA expression profiles of iron-overloaded HT-1080 cells, identifying DE-mRNAs, lncRNAs, and circRNAs with significant upregulation or downregulation alongside their associated biological processes and signaling pathways. Furthermore, circRNA/lncRNA-miRNA-mRNA co-expression network analysis suggested potential regulatory links between iron overload, lysosomal activity, and pathways associated with cancer progression, providing a theoretical framework for future experimental validation. These findings provide an exploratory transcriptomic framework for future functional investigations into ncRNA-mediated regulatory mechanisms in ferroptosis-related cancer biology, while offering transcriptional insights into the interplay between iron overload and lysosomal activity.

## Figures and Tables

**Table 1:** Upregulated DE-mRNAs/lncRNAs/circRNAs.

RNA Type	Gene Name	log_2_ Fold Change	*p* Value	*q* Value
mRNAs	*GRIK2*	13.62	<0.0001	<0.0001
	*AL133500.1*	12.77	<0.0001	<0.0001
	*AL159163.1*	12.69	<0.0001	<0.0001
	*C21orf59-TCP10L*	12.41	<0.0001	<0.0001
	*TMEM189-UBE2V1*	11.98	<0.0001	<0.0001
lncRNAs	*GUSBP1-202*	19.19	<0.0001	0.0269
	*STT3B-202*	17.48	<0.0001	0.0332
	*LINC741*	17.47	<0.0001	0.0240
	*MIR4435-2HG-255*	16.56	<0.0001	0.0282
	*SMYD3-209*	16.35	<0.0001	0.0339
circRNAs	*novel_circ_0001438*	6.84	<0.0001	<0.0001
	*novel_circ_0011056*	5.58	<0.0001	0.0280
	*hsa_circ_0089727*	5.55	<0.0001	0.0280
	*novel_circ_0002937*	5.47	0.0017	0.0349
	*hsa_circ_0001701*	5.45	0.0023	0.0349

**Table 2:** Downregulated DE-mRNAs/lncRNAs/circRNAs.

RNA_Type	Gene_Name	log_2_ Fold Change	*p* Value	*q* Value
mRNAs	*AL121900.2*	−15.23	<0.0001	<0.0001
	*AC117378.1*	−13.74	0.0001	0.0125
	*AC090527.2*	−12.11	<0.0001	<0.0001
	*AC003006.1*	−10.87	<0.0001	0.0090
	*AC104109.3*	−10.73	<0.0001	0.0107
lncRNAs	*ZEB1-223*	−16.86	<0.0001	0.0287
	*AC090809.1-202*	−15.85	<0.0001	0.0012
	*CHCHD6-207*	−15.75	<0.0001	0.0269
	*HIKESHI-206*	−14.95	<0.0001	0.0014
	*PVT1-316*	−14.70	<0.0001	0.0014
circRNAs	*hsa_circ_0004276*	−9.19	0.0187	0.0493
	*hsa_circ_0005785*	−5.59	0.0008	0.0280
	*hsa_circ_0024834*	−5.34	0.0026	0.0349
	*hsa_circ_0005859*	−5.33	0.0024	0.0349
	*hsa_circ_0009000*	−5.29	0.0027	0.0349

## Data Availability

The original contributions presented in this study are included in the article and Supplementary Materials. Further inquiries can be directed to the corresponding author.

## Supplementary Materials

The following supporting information can be downloaded at: https://www.mdpi.com/article/10.3390/ijms27125617/s1.

## References

[1] Bogdan A.R., Miyazawa M., Hashimoto K., Tsuji Y. (2016). Regulators of Iron Homeostasis: New Players in Metabolism, Cell Death, and Disease. Trends Biochem. Sci..

[2] Duca L., Di Pierro E., Scaramellini N., Granata F., Graziadei G. (2025). The Relationship between Non-Transferrin-Bound Iron (NTBI), Labile Plasma Iron (LPI), and Iron Toxicity. Int. J. Mol. Sci..

[3] Ru Q., Li Y., Chen L., Wu Y., Min J., Wang F. (2024). Iron Homeostasis and Ferroptosis in Human Diseases: Mechanisms and Therapeutic Prospects. Signal Transduct. Target. Ther..

[4] Wang S., Ren H., Fan C., Lin Q., Liu M., Tian J. (2024). Ochratoxin a Induces Renal Cell Ferroptosis by Disrupting Iron Homeostasis and Increasing ROS. J. Agric. Food Chem..

[5] Yang Z., Song Y., Li Y., Mao Y., Du G., Tan B., Zhang H. (2022). Integrative Analyses of Prognosis, Tumor Immunity, and ceRNA Network of the Ferroptosis-Associated Gene FANCD2 in Hepatocellular Carcinoma. Front. Genet..

[6] Tang Z., Ye J., Chen D. (2025). HHLA3 Silencing Suppresses KRAS-Mutant Non-Small-Cell Lung Cancer Cell Progression through Triggering MYEOV-Mediated Ferroptosis. J. Biochem. Mol. Toxicol..

[7] Qiu X., Shi Q., Zhang X., Shi X., Jiang H., Qin S. (2022). LncRNA A2M-AS1 Promotes Ferroptosis in Pancreatic Cancer via Interacting with PCBP3. Mol. Cancer Res..

[8] Yamamura S., Imai-Sumida M., Tanaka Y., Dahiya R. (2018). Interaction and Cross-Talk between Non-Coding RNAs. Cell Mol. Life Sci..

[9] Xu H., He Z., Zhang M., Zhou W., Xu C., He M., Wang Z., Wang X. (2023). RNA Seq and ceRNA Network Analysis of the Rat Model of Chronic Kidney Disease. Comb. Chem. High Throughput Screen..

[10] Xia T., Liao Q., Jiang X., Shao Y., Xiao B., Xi Y., Guo J. (2014). Long Noncoding RNA Associated-Competing Endogenous RNAs in Gastric Cancer. Sci. Rep..

[11] Dai N., Ma H., Feng Y. (2023). Silencing of Long Non-Coding RNA SDCBP2-AS1/microRNA-656-3p/CRIM1 Axis Promotes Ferroptosis of Lung Cancer Cells. Cell. Mol. Biol..

[12] Wang M., Mao C., Ouyang L., Liu Y., Lai W., Liu N., Shi Y., Chen L., Xiao D., Yu F. (2019). Long Noncoding RNA LINC00336 Inhibits Ferroptosis in Lung Cancer by Functioning as a Competing Endogenous RNA. Cell Death Differ..

[13] Rockfield S., Raffel J., Mehta R., Rehman N., Nanjundan M. (2017). Iron Overload and Altered Iron Metabolism in Ovarian Cancer. Biol. Chem..

[14] Tian X., Teng Y., Huang M., Zhang Q., Huang J., Chen Y., Wu A., Wang Q., Yu J., Feng J. (2026). MouseOmics: A Multi-Omics Database for Mouse Biological Study. Nucleic Acids Res..

[15] Li H., Wang Y., Chen M., Xiao P., Hu C., Zeng Z., Wang C., Wang J., Hu Z. (2016). Genome-Wide Long Non-Coding RNA Screening, Identification and Characterization in a Model Microorganism Chlamydomonas Reinhardtii. Sci. Rep..

[16] Kopp F., Mendell J.T. (2018). Functional Classification and Experimental Dissection of Long Noncoding RNAs. Cell.

[17] Monteuuis G., Wong J.J.L., Bailey C.G., Schmitz U., Rasko J.E.J. (2019). The Changing Paradigm of Intron Retention: Regulation, Ramifications and Recipes. Nucleic Acids Res..

[18] Yikilmaz E., Rouault T.A., Schuck P. (2005). Self-Association and Ligand-Induced Conformational Changes of Iron Regulatory Proteins 1 and 2. Biochemistry.

[19] Walden W.E., Selezneva A.I., Dupuy J., Volbeda A., Fontecilla-Camps J.C., Theil E.C., Volz K. (2006). Structure of Dual Function Iron Regulatory Protein 1 Complexed with Ferritin IRE-RNA. Science.

[20] Wang C.-Y., Knutson M.D. (2013). Hepatocyte Divalent Metal-Ion Transporter-1 Is Dispensable for Hepatic Iron Accumulation and Non-Transferrin-Bound Iron Uptake in Mice. Hepatology.

[21] Wu C.-S., Lu Y.-J., Li H.-P., Hsueh C., Lu C.-Y., Leu Y.-W., Liu H.-P., Lin K.-H., Huang T.H.-M., Chang Y.-S. (2010). Glutamate Receptor, Ionotropic, Kainate 2 Silencing by DNA Hypermethylation Possesses Tumor Suppressor Function in Gastric Cancer. Int. J. Cancer.

[22] Kuncha S.K., Mazeed M., Singh R., Kattula B., Routh S.B., Sankaranarayanan R. (2018). A Chiral Selectivity Relaxed Paralog of DTD for Proofreading tRNA Mischarging in Animalia. Nat. Commun..

[23] Wang X., Tan X., Zhang J., Wu J., Shi H. (2023). The Emerging Roles of MAPK-AMPK in Ferroptosis Regulatory Network. Cell Commun. Signal.

[24] Liu Y., Liang J., Sha Z., Yang C. (2024). Inhibition of Oxidative Stress-induced Ferroptosis Can Alleviate Rheumatoid Arthritis in Human. J. Immunol. Res..

[25] Branco C.C., Gomes C.T., De Fez L., Bulhões S., Brilhante M.J., Pereirinha T., Cabral R., Rego A.C., Fraga C., Miguel A.G. (2015). Carriers of the Complex Allele HFE c.[187C>G;340+4T>C] Have Increased Risk of Iron Overload in São Miguel Island Population (Azores, Portugal). PLoS ONE.

[26] Memczak S., Jens M., Elefsinioti A., Torti F., Krueger J., Rybak A., Maier L., Mackowiak S.D., Gregersen L.H., Munschauer M. (2013). Circular RNAs Are a Large Class of Animal RNAs with Regulatory Potency. Nature.

[27] Ma Y.-S., Cao Y.-F., Liu J.-B., Li W., Deng J., Yang X.-L., Xin R., Shi Y., Zhang D.-D., Lv Z.-W. (2021). The Power and the Promise of circRNAs for Cancer Precision Medicine with Functional Diagnostics and Prognostic Prediction. Carcinogenesis.

[28] Li X., Yang R., Xu Y., Zhang Y. (2021). Circ_0001438 Participates in the Pathogenesis of Preeclampsia via the Circ_0001438/miR-942/NLRP3 Regulatory Network. Placenta.

[29] Wang L., Ye H., Huang D., Lu C., Lin W., Chen X. (2022). Comprehensive circRNA Analyses in Human Vertebrae of GIOP and Its Molecular Mechanism. Evid. Based Complement. Altern. Med..

[30] Horniblow R.D., Pathak P., Balacco D.L., Acharjee A., Lles E., Gkoutos G., Beggs A.D., Tselepis C. (2022). Iron-Mediated Epigenetic Activation of NRF2 Targets. J. Nutr. Biochem..

[31] Ye Q., Trivedi M., Zhang Y., Böhlke M., Alsulimani H., Chang J., Maher T., Deth R., Kim J. (2019). Brain Iron Loading Impairs DNA Methylation and Alters GABAergic Function in Mice. FASEB J..

[32] Chen P.-H., Tseng W.H.-S., Chi J.-T. (2020). The Intersection of DNA Damage Response and Ferroptosis—A Rationale for Combination Therapeutics. Biology.

[33] Li Y., Jin X., Wang Z., Li L., Chen H., Lin X., Yi S., Zhang Y., Xu J. (2019). Systematic Review of Computational Methods for Identifying miRNA-Mediated RNA-RNA Crosstalk. Brief. Bioinform..

[34] Chan L.S.A., Gu L.C., Wells R.A. (2021). The Effects of Secondary Iron Overload and Iron Chelation on a Radiation-Induced Acute Myeloid Leukemia Mouse Model. BMC Cancer.

[35] Jahng J.W.S., Alsaadi R.M., Palanivel R., Song E., Hipolito V.E.B., Sung H.K., Botelho R.J., Russell R.C., Sweeney G. (2019). Iron Overload Inhibits Late Stage Autophagic Flux Leading to Insulin Resistance. EMBO Rep..

[36] Hu W., Zhou C., Jing Q., Li Y., Yang J., Yang C., Wang L., Hu J., Li H., Wang H. (2021). FTH Promotes the Proliferation and Renders the HCC Cells Specifically Resist to Ferroptosis by Maintaining Iron Homeostasis. Cancer Cell Int..

[37] Zhang Y.-B., Gong J.-L., Xing T.-Y., Zheng S.-P., Ding W. (2013). Autophagy Protein P62/SQSTM1 Is Involved in HAMLET-Induced Cell Death by Modulating Apotosis in U87MG Cells. Cell Death Dis..

[38] Yang M., Chen P., Liu J., Zhu S., Kroemer G., Klionsky D.J., Lotze M.T., Zeh H.J., Kang R., Tang D. (2019). Clockophagy Is a Novel Selective Autophagy Process Favoring Ferroptosis. Sci. Adv..

[39] Zhang R., Liang L., Liao K., Zeng H., Yang X., Wang X., Wang B., Yuan J. (2025). Autophagy Impairment-Derived SQSTM1 Accumulation Promotes Ferroptosis in Corneal Epithelial Cells through ACSL4 in Dry Eye. Investig. Ophthalmol. Vis. Sci..

[40] Wang S., Jiang W., Zhang X., Lu Z., Geng Q., Wang W., Li N., Cai X. (2020). LINC-PINT Alleviates Lung Cancer Progression via Sponging miR-543 and Inducing PTEN. Cancer Med..

[41] Peng X., Yang R., Peng W., Zhao Z., Tu G., He B., Cai Q., Shi S., Yin W., Yu F. (2022). Overexpression of LINC00551 Promotes Autophagy-Dependent Ferroptosis of Lung Adenocarcinoma via Upregulating DDIT4 by Sponging miR-4328. PeerJ.

[42] Lobos-González L., Silva V., Araya M., Restovic F., Echenique J., Oliveira-Cruz L., Fitzpatrick C., Briones M., Villegas J., Villota C. (2016). Targeting Antisense Mitochondrial ncRNAs Inhibits Murine Melanoma Tumor Growth and Metastasis through Reduction in Survival and Invasion Factors. Oncotarget.

