# Supplementary material for: Associative Analysis of lncRNA/circRNA-miRNA-mRNA Expression Profiles in Iron-Overloaded HT-1080 Fibrosarcoma Cells
Source: Int J Mol Sci. 2026 Jun 22;27(12):5617. doi: 10.3390/ijms27125617 (PMC13300622; doi:10.3390/ijms27125617)
Supplement: Supplementary file 1 [file ijms-27-05617-s001.zip › ijms-4218674-supplementary.pdf]

Table S1. Total RNA concentration and integrity detection.

No.	Sample Name	Concentration (ng/μL)	Volume (μL)	Total (μg)	Integrity Value
1	C1	452	32	14.464	9.7
2	C2	526	32	16.832	9.8
3	C3	560	32	17.92	9.7
4	C4	508	32	16.256	9.7
5	C5	457	32	14.624	9.8
6	C6	539	32	17.248	9.9
7	I1	546	32	17.472	9.9
8	I2	516	32	16.512	9.8
9	I3	557	32	17.824	9.6
10	I4	497	32	15.904	9.7
11	I5	505	32	16.16	9.9
12	I6	519	32	16.608	9.5

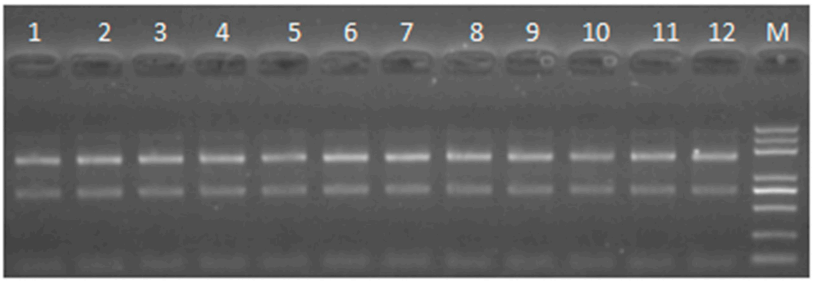

Figure S1. Aarose gel electrophoresis image of RNA samples.

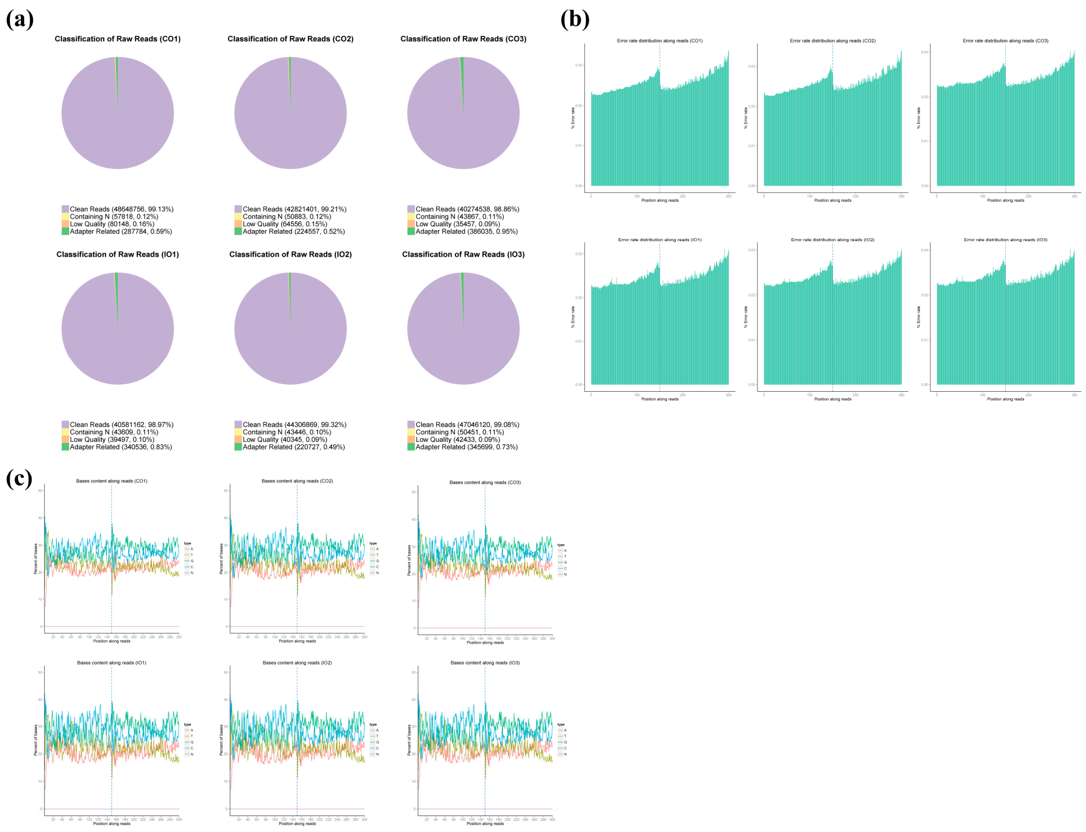

Figure S2. Quality Assessment Diagrams for lncRNA, mRNA, and circRNA Sequencing Data, including all experimental groups (CO1, CO2, CO3, IO1, IO2, IO3). (**a**) Raw data composition. (**b**) Sequencing error rate distribution. (**c**) GC content distribution.

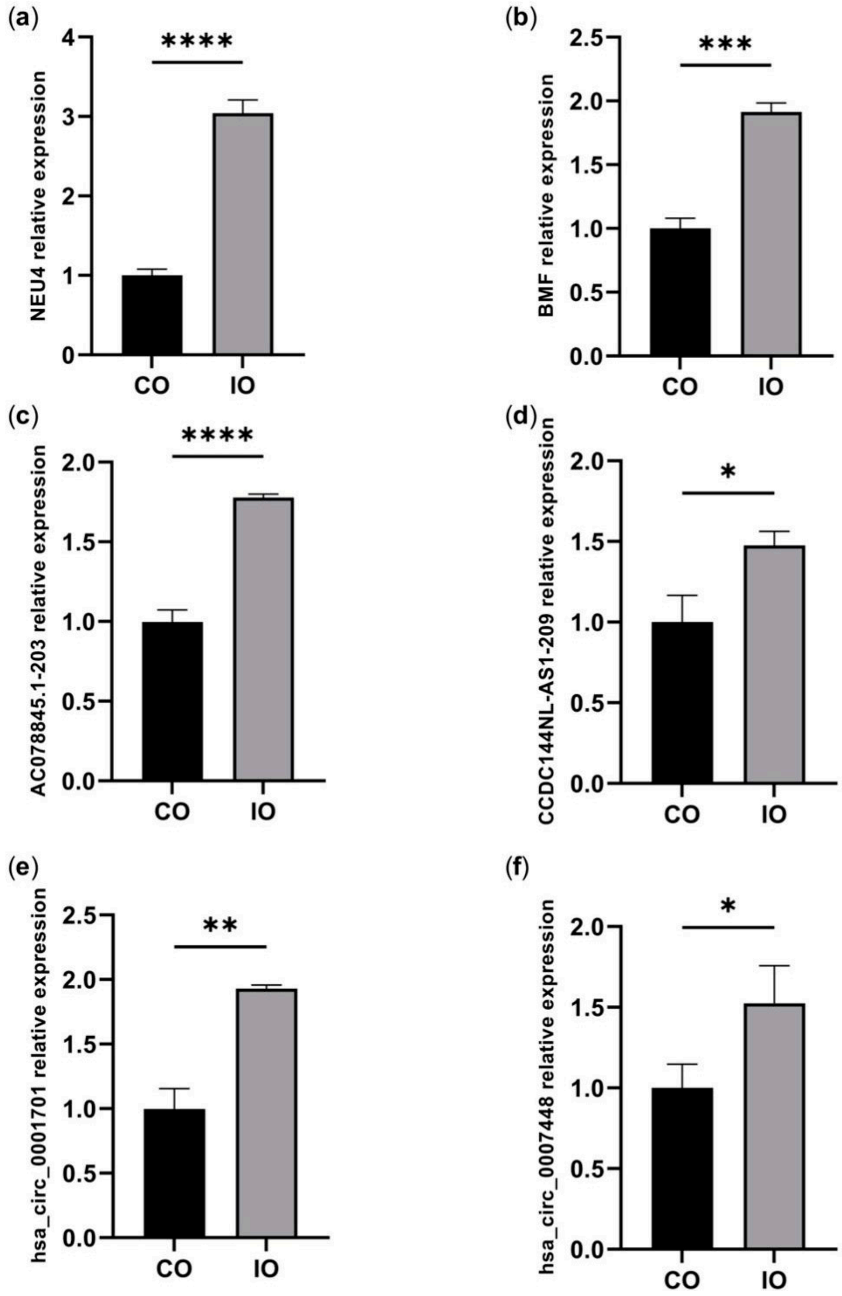

Figure S3. Validation of DE-mRNAs/lncRNAs/circRNAs in HT-1080 cells between the control group (CO) and the iron group (IO) using qRT-PCR. (**a**-**b**) DE-mRNAs: (**a**) NEU4, (**b**) BMF, (**c**-**d**) DE-lncRNAs: (**c**) AC078845.1-203, (**d**) CCDC144NL-AS1-209, (**e**-**f**) DE-circRNAs: (**e**) hsa_circ_0001701, (**f**) hsa_circ_0007448. Data are represented as the mean ± SD (n = 3), * $p < 0.05$, ** $p < 0.01$, *** $p < 0.001$, **** $p < 0.0001$.
